# Septic Pelvic Thrombophlebitis: Diagnosis and Management

**DOI:** 10.1155/IDOG/2006/15614

**Published:** 2006-07-04

**Authors:** Javier Garcia, Ramzi Aboujaoude, Joseph Apuzzio, Jesus R. Alvarez

**Affiliations:** Obstetrics, gynecology, and Women's Health Department, New Jersey Medical School, University of Medicine and Dentistry of New Jersey, Newark, NJ 07103, USA

## Abstract

Septic pelvic thrombophlebitis (SPT) was initially diagnosed and described in the late 1800's. The entity had a high incidence and mortality during this period of time, and a surgical therapeutic approach was the treatment of choice. Since then, the diagnosis, incidence, and management of the entity evolved. This evolution followed the development of newer diagnostic tools such as computed tomography (CT), magnetic resonance imaging (MRI), and a better understanding of the pathophysiology of the disease. The treatment of SPT has had significant changes as well, from a surgical approach at the end of the 19th century to a medical approach after the 1960's. By using an adequate broad-spectrum antibiotic therapy, mortality has decreased. However, controversy in the management of this entity remains even till today.

## BACKGROUND

The entity of septic pelvic thrombophlebitis has evolved profoundly over the last century. The bulk of cases in early reports was mostly obstetric, with a significant number of following abortions. Gynecologic cases with this complication represented the minority.

By the end of the 19th century, von Recklinhausen described an entity in which pelvic infection was characterized by thrombosis of one or both ovarian veins while the remaining pelvis was normal, proposing surgical excision as the therapeutic approach.

The first successful cases of pelvic vein ligation as a treatment for puerperal pyemia were reported between 1898 and 1902 by a number of German physicians including Bumm, Freund, and Trendelenburg [1–3]. In 1951 Collins published his experience with 202 women who underwent surgical intervention and proposed a pathogenic model for suppurative pelvic thrombophlebitis [4, 5].

The incidence of the disease has been dynamic; a clear example is the experience at the Charity Hospital in New Orleans, where between 1941 and 1969, Collins reported a total of 202 cases. A later report (1974–1981) described only three cases in the same institution [4, 5]. This observation was similar to the experience gathered from Grady Memorial Hospital and Emory University Hospital by Josey and Staggers who determined the incidence to be 0.05 percent [6]. In the following decade (1980 to 1989), Dunnihoo et al calculated a similar incidence [1]. Finally, in 1999, Brown and colleagues published a five-year survey of patients delivered at Parkland Hospital finding an overall incidence of 1 : 3000 deliveries. The incidence for vaginal delivery was 1 : 9000 compared to 1 : 800 for cesarean section [3].

Mortality has had a drastic change. Reviews by Williams and Miller reported an average mortality of roughly 50% during the first quarter of the twentieth century in patients undergoing surgery. Collins (1951) reported a 90% survival after the ligation of inferior vena cava and ovarian veins. During the decade of the 1980's, mortality was reported to be 4.4 percent [1].

In 1999 after following a total of 44922 deliveries, 69 patients carried the diagnosis of prolonged infection. Fifteen patients were found to have pelvic thrombophlebitis, however, no complication or death was reported in either group [3].

## CASES

We report two cases which are typical of the eight cases in which the diagnosis of septic pelvic thrombophlebitis was suspected in the past two years. These cases highlight the risk factors that may be associated to the development of septic pelvic thrombophlebitis, the management, and treatment of the entity.

*Case 1*

A 28-year-old primigravid underwent a cesarean section secondary to having a breech presentation and rupture of membranes at 36 weeks gestation. The cesarean section was uncomplicated, but on postpartum day one the patient was having fever and uterine tenderness. A diagnosis of postpartum endometritis was made and the infection was treated with Ertapenem 1 g intravenously daily. After 48 hours of antibiotics, the patient denied uterine tenderness and her WBC count was 12000/mm^3^. Nevertheless, the patient continued to spike fevers and on postpartum day four an abdominal CT scan was obtained. A right ovarian vein thrombosis was noted on the imaging and the patient started therapeutic enoxaparin. After 48 hours of anticoagulation, the patient was afebrile and asymptomatic. The patient was discharged home after being anticoagulated with warfarin and after 6 weeks a CT scan was repeated. The right ovarian thrombosis was not present in the images and warfarin was discontinued.

*Case 2*

A 32-year-old patient underwent a successful in vitro fertilization. At 25 weeks gestation the patient had preterm premature rupture of membranes. The patient was started on penicillin G and a course of steroids was given. After 1 week of expectant management, the patient started to have contractions and a diagnostic amniocentesis was performed. An intraamniotic infection was diagnosed and a classical cesarean section was performed. The patient was started on gentamicin and clindamycin intraoperatively. After 24 hours of antibiotics, the patient continued to have fever and ampicillin was added. On postpartum day 4, the patient complained of pelvic pain and continued to have fever spikes to 101 F. The patient's urinalysis, urine, and blood cultures were negative and her WBC count was 17000/mm^3^. The patient was anticoagulated with enoxaparin after a clinical diagnosis of septic pelvic thrombophlebitis that was suspected and an abdominal CT scan was ordered. The CT scan was negative, but after 24 hours of enoxaparin, the patient became afebrile and her pelvic pain diminished. After 2 days with enoxaparin a WBC count was repeated showing a decrease to 12000/mm^3^. The patient was discharged home on enoxaparin for another week and was followed in clinic for her postoperative appointment. Her pelvic pain was minimal and the rest of her recovery was uncomplicated.

### Pathophysiology

The pathophysiology of pelvic thrombophlebitis as a progression of a pelvic infection was first elucidated by Collins and later Duff, Gibbs, and others [1, 4, 5].

The pathogenesis is thought to include injury to the intima of the pelvic vein caused by a spreading uterine infection, bacteremia, and endotoxins, which can also occur secondary to the trauma of delivery or surgery. In this setting, Virchow's triad is completed due to the contribution of pregnancy as a well-known hypercoagulable state, and the reduction of blood flow in dilated uterine and ovarian veins during the postpartum period which causes venous stasis. Phlebographic studies have demonstrated a left to right venous flow in upright position, which may explain the dextro-prevalence of ovarian vein thrombosis [1].

### Diagnostic approach

A suspicion of SPT should arise when fever, which usually follows a spiking pattern, fails to respond to standard broad-spectrum antibiotic therapy. Septic pelvic thrombophlebitis was diagnosed in 20 percent of patients with prolonged febrile morbidity, defined as more than five days of fever regardless of appropriate antimicrobial treatment [3].

Patients often complain of flank and lower abdominal pain, typically described as noncolicky and constant. Pain may be of variable intensity and may radiate to the groin or upper abdomen, and paralytic ileus may occur.

Upon physical examination, the patient usually does not appear toxic, there may be tenderness to palpation in the lower abdomen, and an occasional tender abdominal mass described as rope or sausage-shape may be identified. This represents the most diagnostic finding in an abdominal exam, but it is rare.

Other clinical characteristics of septic pelvic thrombophlebitis have changed over time. Pulmonary emboli, was considered a criterion for diagnosis of the condition. Initial reports described pulmonary embolic phenomena to be frequent, small, and multiple [4, 5, 7].

During the decades of the 1960's through 1970's, some authors reported that 32 to 38 percent of patients with septic pelvic thrombophlebitis had evidence of pulmonary emboli [7]. Most of these diagnoses were made only on the basis of radiographic findings. However, other contemporary investigators demonstrated an incidence of pulmonary embolism in a range of 2.7%. The latest series (1999) did not report a single embolic event after following up 44922 patients over a 3-year period [3]. Refinements in the diagnostic methodology as well as implementation of earlier and more effective treatment are likely the reasons responsible for the remarkable decline in the incidence of embolic events.

Diagnostic tools like computed tomography (CT) and magnetic resonance imaging (MRI) are used to provide confirmation of pelvic thrombophlebitis [1]. Tomographic criteria for diagnosis include (1) enlargement of the vein involved, (2) low density lumen within the vessel wall, and (3) sharp enhancement of the vessel wall. When using MRI the thrombosed vessel will appear bright whereas normal blood flow looks dark.

It is believed that both methods are comparable. MRI offers a better visualization of soft tissue changes, being able to evaluate the resolution of edema and inflammatory signs. However, both techniques have their limitations related to visualizing smaller vessels such as uterine, cervical, and other smaller pelvic branches.

Anecdotal experiences with pelvic ultrasound used for the diagnosis of ovarian vein thrombosis revealed a limited utility; nevertheless, ultrasonography may have a role in monitoring treatment response [1].

The only laboratory test that may aid in the diagnosis and management of the disease is a complete blood count with blood cultures. Nevertheless, blood cultures provide identification of a microorganism in less than 35 percent of the cases [1, 4, 5].

### Management and treatment

By the turn of the century, surgical excision of the thrombosed vein was the treatment of choice [1]. After publishing the experience at the Charity Hospital in New Orleans in 1951, Collins advocated the ligature of the inferior vena cava and ovarian veins as the optimal treatment reporting an improvement in mortality rates after the procedure.

During the late sixties, heparin anticoagulation in conjunction with antibiotic coverage gained general acceptance and proved to be safe. These reports were observational, patient samples were small and heterogeneous, and the diagnosis of pelvic septic thrombophlebitis was presumptive in most of the cases [7].

The absence of a definitive diagnostic technique made it difficult to corroborate a clinical suspicion and compare the effectiveness of treatment regimens proposed.

It was believed that the diagnosis of septic pelvic thrombophlebitis depended on a rapid return to normal temperature after heparin was given, an observation that is currently being questioned.

In the last 20 years, the introduction of computed tomography and magnetic resonance imaging has revolutionized the diagnosis of pelvic thrombophlebitis allowing investigators to assess response to heparin therapy. Several observational studies questioning the efficacy of anticoagulation were followed by Brown and associates, who prospectively gathered a total of 14 cases over a three-year period at Parkland Hospital (Dallas); one group of patients was randomized to antimicrobial therapy with the addition of heparin, others were assigned to antibiotics alone [6]. Duration of fever and hospital stay was similar in both groups. Furthermore, neither thromboembolic events nor reinfection were reported [3]. The Brown et al study does not support the empiric use of anticoagulants for SPT.

Antibiotic management follows the experience of Di Zerega et al (1979), who determined that response to Clindamycin and Gentamycin in women with endometritis following a cesarean delivery was adequate [8]. Since then, many comparative studies with novel single and multiagent antibiotic therapies failed to demonstrate superior efficacy. In 1988 Walmer et al associated failure of the Clindamycin-Gentamycin combination with enterococcal infection. Addition of ampicillin resulted in the “triple antibiotic regimen” model. Most institutions recommend this regimen for treatment of puerperal infections; however, if fever persists after 5 days of antibiotics, it is suggested to use an alternative antibiotic agent. Imipenem or ertapenem, with its broad spectrum and anti-pseudomonal activity, represent a reasonable selection.

The incidence of septic pelvic thrombophlebitis is likely to rise, as the cesarean section rates continue to climb. It is still recommended to treat this entity with broad spectrum antibiotics. Nevertheless, there are no recommendations available to guide physicians in regards to anticoagulation therapy. Only heparin use has been described but is controversial as seen by Brown et al study [3]. However, for those who prescribe anticoagulant therapy, can low molecular weight heparin anticoagulation therapy be used instead of unfractionated heparin? How long should the patient be anticoagulated? Is there a role for anticoagulation with oral warfarin? Is there a role of anticoagulation therapy in the management of septic pelvic thrombophlebitis? These are the questions for which there are no answers yet and further randomized studies should be done.

In our retrospective review, we have had the experience of successfully managing eight cases of septic pelvic thrombophlebitis over the last 2 years. We used anticoagulation therapy in all cases in addition to broad spectrum antibiotic therapy. When patients did not respond to broad spectrum antibiotics after 5 days, SPT was suspected, anticoagulation was initiated with subcutaneous enoxaparin and a CT scan or MRI of the pelvis was ordered. If the imaging showed a small pelvic thrombosed vessel, therapeutic enoxaparin would be continued for 2 weeks. If the pelvic imaging was negative, then therapeutic enoxaparin was given only for 1 week. The only instance were a patient was converted to oral warfarin and maintained in therapeutic dosage for 3 months was if a right ovarian vein thrombosis was seen. A follow up with pelvic MRI was done 3 months after treatment. The cases that we managed fit into the three categories described in Table 1.

## Figures and Tables

**Table 1:** Diagnosis and management for presumed septic pelvic thrombophlebitis.

CT or MRI findings	Antibiotics	Anticoagulation	Length of treatment	Final outcome

Right ovarian vein thrombosis	Ertapenem or gentamicin, ampicillin, clindamycin (7 days)	Enoxaparin (1 mg/Kg), warfarin (INR 2.5)	3–6 months warfarin	Repeat CT scan after 3 months. If negative, stop anticoagulation. If still positive for thrombi, anticoagulate for 3 additional months

Pelvic branch vein thrombosis	Ertapenem or gentamicin, ampicillin, clindamycin (7 days)	Enoxaparin (1 mg/Kg)	2 weeks	No repeat imaging necessary

Negative for pelvic thrombi	Ertapenem or gentamicin, ampicillin, clindamycin (7 days)	Enoxaparin (1 mg/Kg)	1 week	No repeat imaging necessary
